# Borrelia burgdorferi infections in children and adolescents in Switzerland – a seroprevalence study 2023/2024 (BOBUINCA)

**DOI:** 10.1007/s15010-024-02387-7

**Published:** 2024-09-26

**Authors:** Laura Heeb, Nora Fritschi, Andrea Marten, Tatjana Welzel, Nicole Ritz, Ulrich Heininger

**Affiliations:** 1 Infectious Disease and Vaccinology Unit, University Children’s Hospital Basel, University of Basel, Spitalstrasse 33, Basel, CH-4031 Switzerland; 2 Department of Clinical Research, University of Basel, Basel, Switzerland; 3 Department of Paediatrics and Paediatric Infectious Diseases, Children’s Hospital of Central Switzerland, Lucerne, Switzerland; 4 Pediatric Rheumatology, University Children’s Hospital Basel, University of Basel, Basel, Switzerland; 5 Pediatric Research Center, University Children’s Hospital Basel, University of Basel, Basel, Switzerland; 6 Department of Paediatrics, The Royal Children’s Hospital Melbourne, The University of Melbourne, Melbourne, Australia

**Keywords:** Lyme borreliosis, Seroprevalence, Paediatric, Switzerland, Tick

## Abstract

**Background:**

Lyme borreliosis is one of the most prevalent tick-borne diseases in Europe. Studies on seroprevalence of Borrelia burgdorferi IgG antibodies in children are rare. The aim of this study was to determine the seroprevalence of B. burgdorferi IgG antibodies in children and adolescents residing in North-Western Switzerland and neighbouring countries.

**Methods:**

Prospective cross-sectional observational single-centre study using left-over plasma of asymptomatic paediatric patients. Included were children aged 1–17 years living in North-Western Switzerland and bordering areas of France and Germany. Excluded were children with symptoms of Lyme borreliosis or a chronic disease possibly affecting plasma antibodies (immunodeficiency syndrome, systemic lupus erythematosus) or with such medication (e.g., intravenous immunoglobuline treatment, allogenic stem cell transplantation, immunosuppressive treatment) as well as refugees seeking asylum. IgG antibodies against B. burgdorferi were measured by ELISA and positive or borderline results by line blot. Positivity was defined as scenario 1: ELISA positive/line blot positive or borderline OR ELISA borderline/line blot positive. Scenario 2: ELISA positive or borderline/line blot positive. A multivariable logistic regression model for seropositivity was applied.

**Results:**

962 children were included (mean age 9.63 years, standard deviation 5.01, 54.5% males). Seroprevalence for scenario 1 was 13.3% (95% CI: 11.2–15.6) and for scenario 2 11.2% (95% CI: 9.3–13.4). Seroprevalence (scenario 1) was comparable for age groups, sex and rural versus urban residence.

**Conclusion:**

This study shows an increased seroprevalence for B. burgdorferi in the paediatric age compared to previous childhood studies. We also found an increased risk for B. burgdorferi infection at young age.

## Introduction

Lyme borreliosis has the highest seroprevalence of all tick-borne disease in Europe and is caused by spirochetes of the *Borrelia (B.) burgdorferi sensu lato* complex. Its clinical presentation varies greatly depending on the stage of disease: in children and adolescents, the localised stage presents with erythema migrans or borrelial lymphocytoma, the early disseminated stage with *neuroborreliosis*, acute arthritis or Lyme carditis, and the late disseminated stage with Lyme arthritis, less often as acrodermatitis chronica atrophicans or late neuroborreliosis [1, 2]. The great majority of infections, however, are asymptomatic [3].

The worldwide highest seroprevalence of *B. burgdorferi* infections has been found in Europe with 20.7% (95% Confidence Interval (CI): 13.8–28.6), as demonstrated in a recently published systematic review and meta-analysis [4]. Most of the recent studies on *B. burgdorferi* seroprevalence in Europe have been done in adults with values in the range of 3.9% (95% CI: 3.0-5.1) in Finland [5], 4.4% (95% CI: 3.5–5.2) in the Netherlands [6] to 13.01% in Slovakia [7].

There are only few studies on *B. burgdorferi* seroprevalence in children and adolescents in Europe. In Sweden in 2010, seroprevalence of *B. burgdorferi* IgG (Immunoglobulin G) in 2’000 children aged 5 years was 3.2% [8]. In a German study in 12’614 children and adolescents 1–17 years of age in 2012, seroprevalence was 4.0% (95% CI: 3.6–4.5) [9]. This result was confirmed in the most recent study in the paediatric age group in Germany in 2023 with a seroprevalence of 4.1% (95% CI: 3.2–5.1) in 2’891 participants [10]. In both studies seroprevalence increased with age, was higher in male individuals and in southern [9] and south-eastern regions compared to the rest of Germany [10], respectively. Children appear to be at a particularly high risk of *B. burgdorferi* infections, as multiple studies have shown higher rates among individuals < 20 years of age and seniors, i.e. >60 years of age compared to other age groups [11] with peaks of seroprevalence in the 5–10 year and 45–70 year age cohorts [12].

*B. burgdorferi* infections are not notifiable in Switzerland. However, reporting of clinical manifestation, i.e. Lyme borreliosis, is done on a voluntary basis through the so-called Sentinella programme of the Swiss Federal Office of Public Health [13]. Yet, the actual infection rate cannot be inferred from incidence of the disease. Interpretation of *B. burgdorferi* serology as a diagnostic test in symptomatic children and adolescents is challenging when the local seroprevalence and thus pre-test probability (i.e., positive and negative predictive values) is unknown. Therefore, knowing the age-dependent seroprevalence of *B. burgdorferi* infections and its associated risk factors will help health care professionals with the interpretation of serological findings in patients with suspected Lyme borreliosis.

The aim of this study was to evaluate the seroprevalence of *B. burgdorferi* IgG antibodies in children residing in North-Western Switzerland and bordering areas of France and Germany without current symptoms of Lyme borreliosis.

## Methods

### Study design, setting and population

This was a cross-sectional, observational, single-centre study with further use of data and left-over plasma in children cared at the University Children`s Hospital Basel. The University Children’s Hospital Basel is providing secondary and tertiary care for children and adolescents in North-Western Switzerland and in addition partly for the neighbouring regions of France and Germany.

We included specimens from patients 1 to 17 years of age residing in the Swiss cantons Basel-Land (country), Basel-Stadt (city), Aargau, Solothurn, Jura and bordering areas of France and Germany with documented agreement to the further use of health-related data and left-over material for research purposes in the general consent. Children presenting with symptoms of Lyme borreliosis and refugees seeking asylum were excluded. Furthermore, we excluded patients with a chronic underlying disease or medication possibly affecting plasma antibodies such as known inborn or acquired immunodeficiency syndrome, systemic lupus erythematosus, history of intravenous immunoglobulin treatment in the past 12 months for any reason including Kawasaki Disease, Paediatric Inflammatory Multisystem Syndrome (PIMS) or immune thrombocytopenia, patients after allogenic stem cell transplantation. with cancer or known chronic haematologic disease, treated with immunosuppressive treatment in the last 6 months including systemic steroids > = 2 weeks duration ( > = 2 mg/kg or > = 20 mg prednisone equivalent), immunosuppressive combination treatments (e.g. biological disease modifying antirheumatic drugs (DMARDs) + conventional DMARDs), Rituximab or leflunomid.

Left-over EDTA (Ethylendiamintetraacetat) blood sample specimens from in- and out-patients were included between June 26, 2023, and February 9, 2024. If a patient had blood taken more than once during the study period, only the first specimen was included. All samples were obtained for indicated clinical tests and stored at the haematology laboratory at room temperature for three days before release for study purposes. Plasma-separation was done by centrifugation for 10 min at 4 °C at 2’800 G and stored in aliquots of 25 µL in a freezer at -75 °C until batched analysis.

### Serological assays

*B. burgdorferi* IgG antibodies were determined in a two-tier testing. First, *B. burgdorferi* IgG ELISA (Enzyme-linked Immunoassay) test was done (first aliquot, Borrelia + VsIE IgG ELISA, TECAN, Männedorf, Switzerland, RE57201). Second, in case of a positive or borderline ELISA test result, a line blot was performed (Borrelia EU + TpN17 IgG, Gold Standard Diagnostics, Dietzenbach, Germany).

ELISA and line blot were done and interpreted according to the manufacturer’s instructions. In the ELISA analysis, *B. burgdorferi* IgG antibody concentrations of 9–11 U/mL were classified as “borderline” and a concentration of *≥* 12 U/mL as “positive”. In the line blot analysis, one visible line was classified as “borderline” and at least two lines as “positive”.

Since there is potential serologic cross reaction between antibodies against *B. burgdorferi* and *Treponema pallidum*, the line blot not only detects antibodies against *B. burgdorferi* specific antigens but also against Treponema pallidum antigen 17 (TpN17). If the line blot was positive for TpN17, a specific serologic test for *Treponema pallidum* was performed (LIAISON^®^ Treponema Screen, an automated chemiluminescence immunoassay (CLIA), DiaSorin, Saluggia, Italy) to confirm or rule out syphilis.

To determine the specificity of the ELISA, a subset of ELISA-negative specimens (matched with positive specimens by age, sex, collection date and chronic disease status) was tested by line blot. We then extrapolated the line blot results of the tested to the total negative ELISA samples.

### Collected variables

The following information was collected from electronic patient records: demographic data such as age, sex and place of residency (country and postal code) as well as clinical information such as reason for consultation, in- / outpatient visit, underlying diseases, and previous / ongoing treatment. Rural residence was defined as population density < 300 inhabitants/km^2^ and urban residence as population density ≥ 300 inhabitants/km^2^.

### Bias

A possible selection bias could have occurred as patients with a chronic disease are likely overrepresented in our study due to collection of specimens in a hospital laboratory setting. They may spend less time outdoors with a decreased risk of tick bites which could have led to an underestimation of the seroprevalence in the general same age population, extrapolated from our study. However, as most of the included patients had non-serious diseases (e.g. asthma), we do not consider this bias to be strong. To minimise these potential selection biases, we conducted a subgroup analysis comparing the seroprevalence in children with and without chronic diseases.

### Statistical analysis

**Fig. 1:**
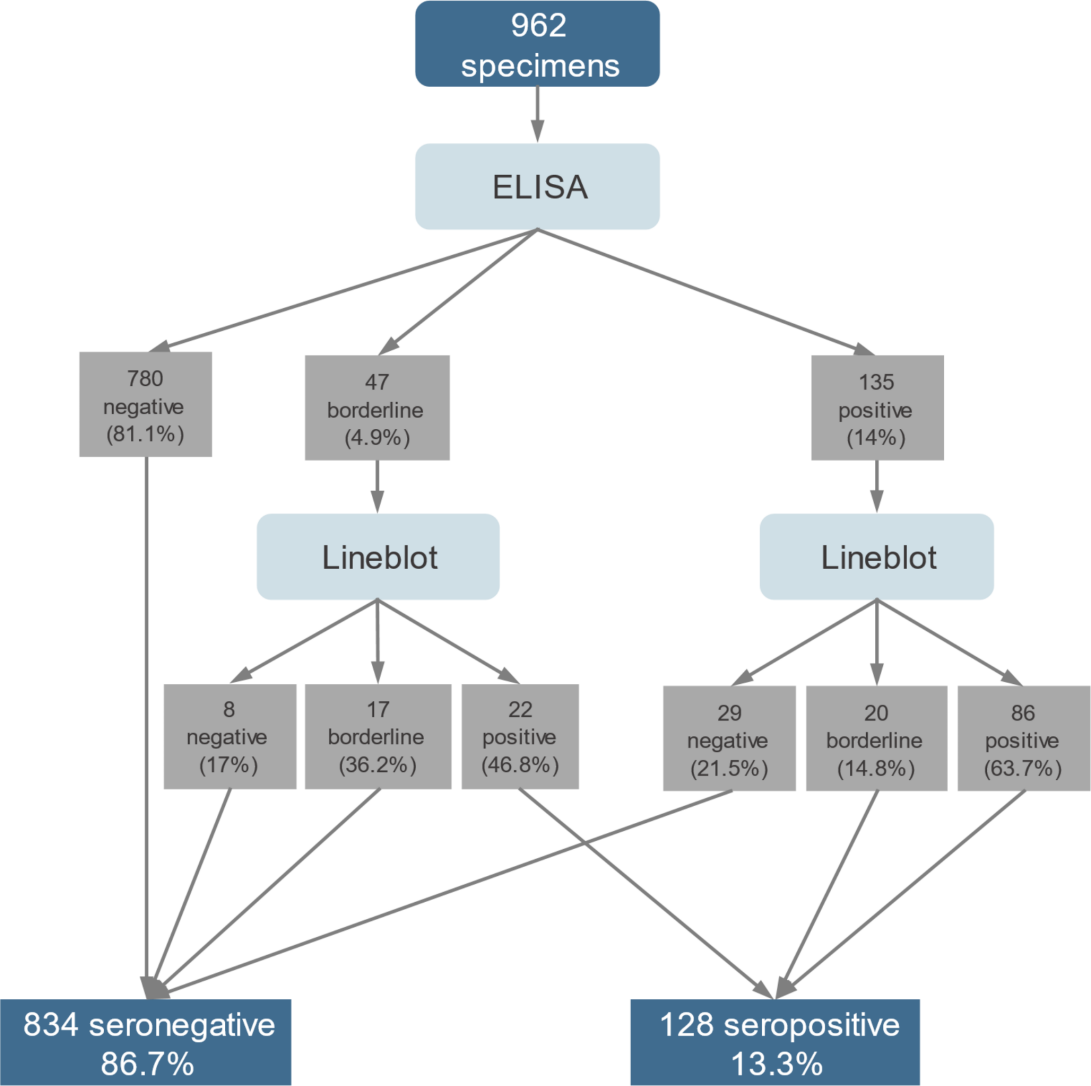
Two-tier testing of scenario 1 for determination of *B. burgdorferi* IgG antibodies in a cross-sectional study of children, Switzerland, 2023–2024 (*n* = 962)

**Fig. 2:**
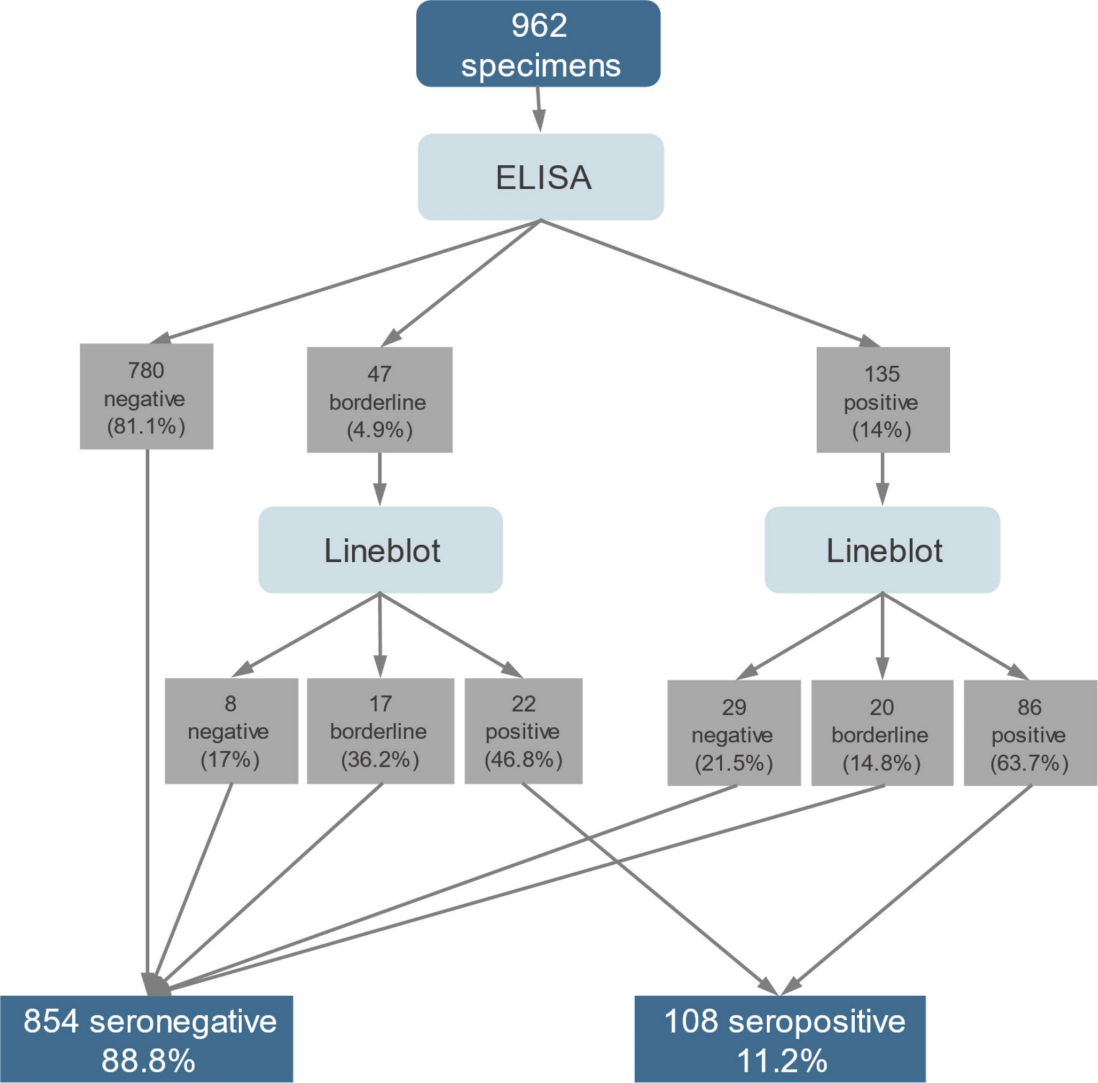
Two-tier testing of scenario 2 for determination of *B. burgdorferi* IgG antibodies in a cross-sectional study of children, Switzerland, 2023–2024 (*n* = 962)

We hypothesised that the overall seroprevalence has increased in comparison to former studies and that a higher seroprevalence can be observed with increasing age. The calculation of seroprevalence was done for two different scenarios:

- Scenario 1: ELISA positive/line blot positive or borderline OR ELISA borderline/line blot positive (Fig. 1).
- Scenario 2: ELISA positive or borderline/line blot positive (Fig. 2).

Pearson’s chi-squared test was used to assess differences for categorical data and t-test for continuous data. A permutation test was used to assess differences in the proportion of seropositivity in different collection months. Comparisons of baseline characteristics (Table 1) with chi-squared and t-test, permutation as well as logistic regression models were based on seropositivity definition according to scenario 1.

**Table 1:** Serostatus of *B. burgdorferi* IgG antibodies by specific characteristics

	Negative * (*n* = 834)	Positive * (*n* = 128)	Total (*N* = 962)	*p*-value
**Sex**				0.788
Male	452 (86.4%)	71 (13.6%)	523 (100.0%)	
Female	382 (87.0%)	57 (13.0%)	439 (100.0%)	
**Age (in years)**				0.436
Mean (SD)	9.577 (5.016)	9.948 (4.991)	9.627 (5.012)	
Range	1.000–17.917	1.000–17.917	1.000–17.917	
**Age groups (in years)**				0.975
1-4.5	175 (87.5%)	25 (12.5%)	200 (100.0%)	
> 4.5 < 8	167 (86.1%)	27 (13.9%)	194 (100.0%)	
8 < 12	169 (87.6%)	24 (12.4%)	193 (100.0%)	
12 < 15	168 (86.6%)	26 (13.4%)	194 (100.0%)	
15 < 18	155 (85.6%)	26 (14.4%)	181 (100.0%)	
**Residency** ^1^				0.57
AG	72 (81.8%)	16 (18.2%)	88 (100.0%)	
BL	340 (86.1%)	55 (13.9%)	395 (100.0%)	
BS	282 (87.0%)	42 (13.0%)	324 (100.0%)	
JU	26 (89.7%)	3 (10.3%)	29 (100.0%)	
SO	73 (91.2%)	7 (8.8%)	80 (100.0%)	
France	8 (80.0%)	2 (20.0%)	10 (100.0%)	
Germany	33 (91.7%)	3 (8.3%)	36 (100.0%)	
**Collection month**				0.015
06/2023	13 (86.7%)	2 (13.3%)	15 (100.0%)	
07/2023	62 (92.5%)	5 (7.5%)	67 (100.0%)	
08/2023	99 (90.0%)	11 (10.0%)	110 (100.0%)	
09/2023	96 (85.7%)	16 (14.3%)	112 (100.0%)	
10/2023	143 (90.5%)	15 (9.5%)	158 (100.0%)	
11/2023	112 (78.3%)	31 (21.7%)	143 (100.0%)	
12/2023	117 (81.8%)	26 (18.2%)	143 (100.0%)	
01/2024	153 (90.5%)	16 (9.5%)	169 (100.0%)	
02/2024	39 (86.7%)	6 (13.3%)	45 (100.0%)	
Chronic disease **				0.047
No	351 (89.3%)	42 (10.7%)	393 (100.0%)	
Yes	483 (84.9%)	86 (15.1%)	569 (100.0%)	
**Type of visit**				0.189
Inpatient	227 (84.4%)	42 (15.6%)	269 (100.0%)	
Outpatient	607 (87.6%)	86 (12.4%)	693 (100.0%)	
**Population density**				0.122
Rural	413 (88.4%)	54 (11.6%)	467 (100.0%)	
Urban	415 (85.0%)	73 (15.0%)	488 (100.0%)	

*Using definition of scenario 1: ELISA positive/line blot positive or borderline OR ELISA borderline/line blot positive

**Defined as a disease that requires regular monitoring or therapy

^1^ Cantons of Switzerland: AG Aargau, BL Basel-Land, BS Basel-Stadt, JU Jura, SO Solothurn

Age stratified analysis was done in the following age groups: 1-4.5 years, > 4.5 < 8years, 8 < 12 years, 12 < 15 years, 15 < 18 years.

The postal code was used to determine the population density, which was determined based on data obtained from the Federal Statistical Office of the respective country [14–18]. The variables age, sex, chronic disease, residency, collection month, urban/rural were evaluated as predictor in univariable logistic regression models for seropositivity as outcome. If a significance level of α < 0.2 was met, the variable was included in a multivariable logistic regression model along with the variables age and sex, which were included a priori. The significance level was set at *p* < 0.05. For proportions the 95% CI was calculated by the method of Clopper and Pearson. For calculation of sensitivity and specificity, all borderline test results were excluded. Since only about one quarter of the ELISA negative results were tested by line blot, the findings were extrapolated to the total ELISA negative study population. Statistical analyses were done using R and R Studio (R Version 4.3.2).

## Results

### Study population

During the study period, a total of 8’893 blood specimens were taken from 4’479 patients. These patients were screened during the occasion of obtaining their first blood specimen and 3501 (78.2%) patients were not eligible for participation for the following reasons: lack of informed consent (*n* = 2374, 67.8%), lack of informed consent and age < 1 year (*n* = 713, 20.4%), informed consent but age < 1 year (*n* = 129, 3.7%), and various combinations of other exclusion criteria including <10 patients with a known previous history of Lyme borreliosis (*n* = 285, 8.1%). The remaining 978 patients were included in the study. Blood volume of specimens of 16 patients was too small for serologic testing, resulting in 962 specimens for study analysis.

### Descriptive data

Patient mean age was 9.63 (SD 5.01, median 9.25, IQR: 5.08–14.25) years (10.37 in females vs. 9.00 years in males; *p* < 0.001). Overall, 524 (4.5%) patients were male and 438 (45.5%) were female. Most patients resided in the cantons Basel-Land (*n* = 395, 41.1%), Basel-Stadt (; *n* = 324, 33.7%), and Aargau (88, 9.1%). Similar proportions lived in urban (*n* = 488, 51.1%) and rural (*n* = 467, 48.9%) areas (*n* = 7 unknown). The majority of patients had underlying chronic diseases (*n* = 569, 59.1%). Further details are presented in Table 1.

ELISA analysis for *B. burgdorferi* IgG antibodies showed that 780 samples (81.1%) were negative, 135 (14%) positive and 47 (4.9%) borderline results. Line blot analysis of ELISA positive results showed 86 (63.7%) positive and 20 (14.8%) borderline results. Line blot analysis of 47 ELISA borderline results showed 22 (46.8%) positive. Based on the definition of seropositivity for scenario 1, 128 samples were considered seropositive resulting in a seroprevalence of 13.3% (95% CI: 11.2–15.6) (Fig. 1). Based on the definition for seropositivity in scenario 2, 108 were seropositive resulting in a seroprevalence of 11.2% (95% CI: 9.3–13.4, Fig. 2).

**Fig. 3:**
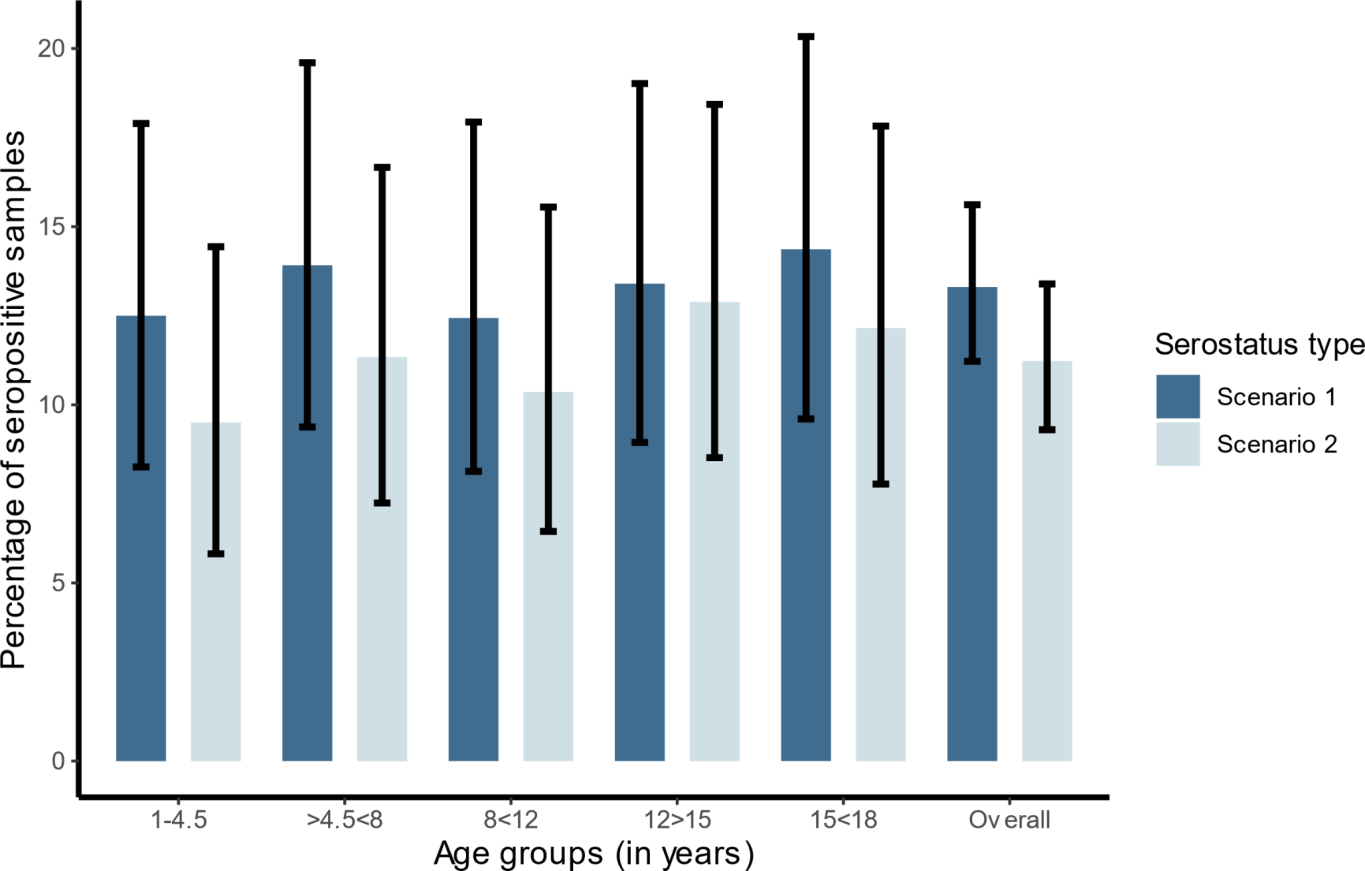
Seroprevalence of *B. burgdorferi* IgG antibodies by age groups in a cross-sectional study of children and adolescents, Switzerland, 2023–2024 (*n* = 962)*. * Scenario 1: ELISA positive/line blot positive or borderline OR ELISA borderline/line blot positive. Scenario 2: ELISA positive or borderline/line blot positive. A multivariable logistic regression model for seropositivity was applied

Further analyses were done according to scenario 1. In scenario 1 seroprevalence was comparable in all age group (Fig. 3). The seroprevalence was similar in both sexes with 13.6% (95% CI: 10.8–16.8) for males and 13.0% (95% CI: 10-16.5) for females (*p* = 0.77). In the cantons Aargau (18.2%), Basel-Land (13.9%) and Basel-Stadt (13%) the calculated seroprevalence proportions were higher than in Solothurn (8.8%) but this difference was not statistically significant (*p* = 0.57). Seroprevalence in patients residing in urban areas was similar to those in rural regions (15% vs. 11.6%, *p* = 0.12) but it was higher in patients with chronic disease compared to those without (14.8% vs. 10.4%, *p =* 0.047).

**Fig. 4:**
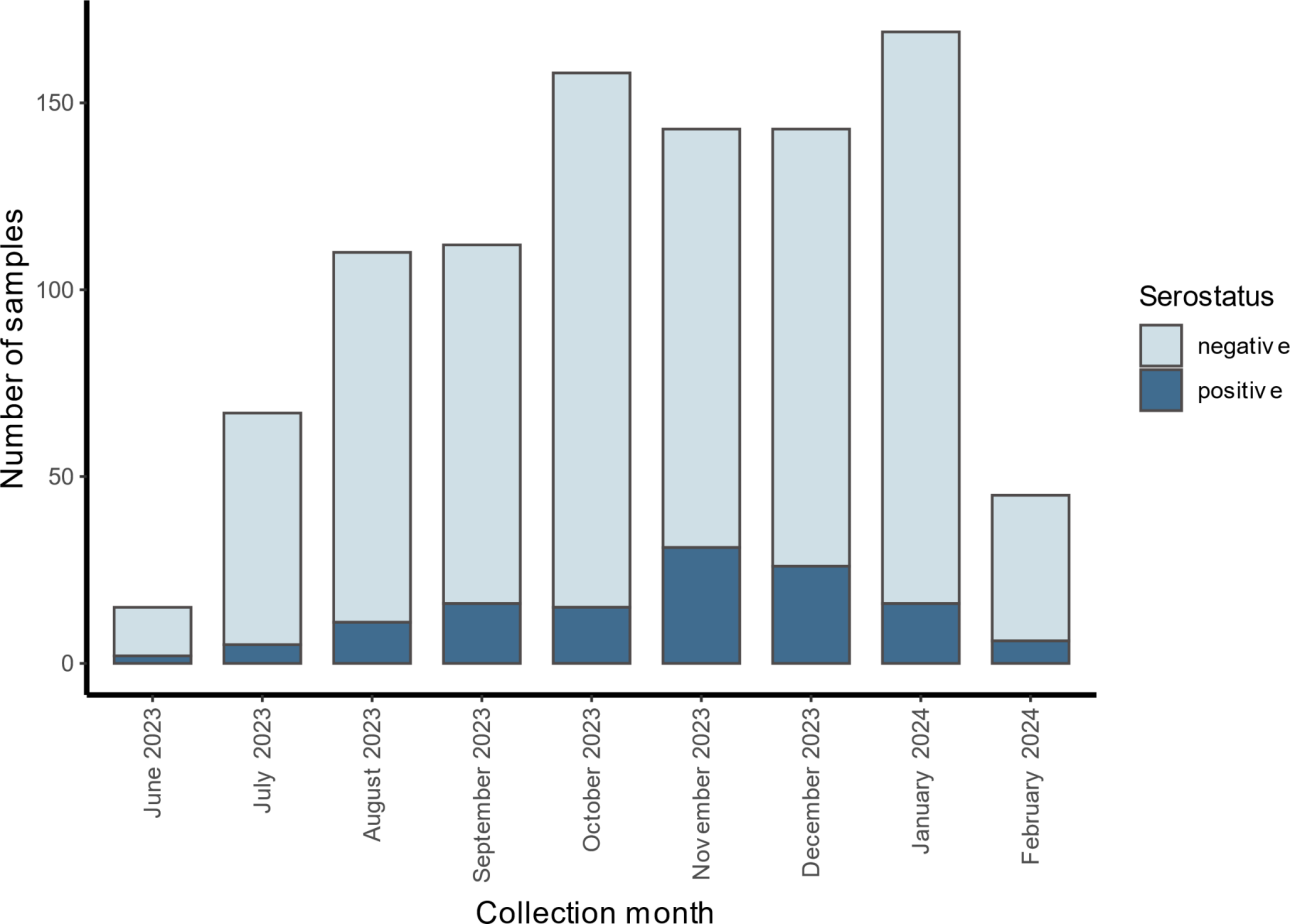
Serostatus of *B. burgdorferi* IgG antibodies by collection month in a cross-sectional study of children, Switzerland, 2023–2024 (*n* = 962)

We found a tendency of higher seroprevalence in specimens obtained during the months of November (21.7%) and December (18.2%) compared to all other months of the study period. However, the seropositivity proportion of 21.7% in November was not significantly higher than in the other months (*p =* 0.21, 10’000 permutations for months tested) (Fig. 4).

### Logistic regression models

The odds for seropositivity were significantly increased for region of residency, chronic disease, collection month and population density in the univariable logistic regression models (Table 2). In a multivariable logistic regression model for seropositivity only collection of samples in the months of November (OR (Odds ratio) = 2.43, *p* = 0.01) and December (OR = 2.25, *p* = 0.02) was significantly increased after correction for age, sex, chronic disease, residency, collection month, urban/rural.

**Table 2:** Results of multivariable logistic regression analysis of the seroprevalence of *B. Burgdorferi* IgG antibodies*

	Univariable log regression			Multivariable log regression		
	OR	95% CI	*p*-value	OR	95% CI	*p*-value
**Sex**						
*Male*			*Ref.*			*Ref.*
Female	1.03	[0.71, 1.49]	0.89	0.96	[0.65, 1.42]	0.83
**Age (in years)**	1.01	[0.98, 1.05]	0.44	1.02	[0.98, 1.06]	0.39
**Collection month**						
*January*			*Ref.*			*Ref.*
February	1.47	[0.5, 3.84]	0.45	1.15	[0.35, 3.16]	0.8
June	1.47	[0.22, 5.98]	0.63	1.62	[0.24, 6.71]	0.55
July	1.47	[0.22, 5.98]	0.63	0.77	[0.24, 2.07]	0.62
August	1.06	[0.46, 2.36]	0.88	1.09	[0.47, 2.45]	0.83
September	1.59	[0.76, 3.36]	0.22	1.53	[0.71, 3.28]	0.27
October	1	[0.47, 2.11]	0.99	0.92	[0.43, 1.98]	0.83
November	2.65	[1.4, 5.18]	0	2.41	[1.25, 4.8]	0.01
December	2.12	[1.1, 4.22]	0.03	2.25	[1.15, 4.5]	0.02
Chronic disease**						
*No*			*Ref.*			*Ref.*
Yes	1.49	[1.01, 2.22]	0.05	1.51	[1, 2.31]	0.05
**Residency** ^1^						
*AG*			*Ref.*			*Ref.*
BL	0.73	[0.4, 1.38]	0.31	0.82	[0.44, 1.61]	0.55
BS	0.67	[0.36, 1.29]	0.21	0.65	[0.34, 1.31]	0.21
Jura	0.52	[0.11, 1.72]	0.33	0.7	[0.15, 2.42]	0.6
SO	0.43	[0.16, 1.08]	0.08	0.47	[0.17, 1.22]	0.13
France	1.13	[0.16, 5.03]	0.89	0.88	[0.12, 4.22]	0.88
Germany	0.41	[0.09, 1.33]	0.18	0.34	[0.05, 1.33]	0.17
**Population density**						
Rural			*Ref.*			*Ref.*
Urban	1.35	[0.92, 1.97]	0.12	1.29	[0.86, 1.95]	0.22

* Using definition of scenario 1: ELISA positive/line blot positive or borderline OR ELISA borderline/line blot positive

** Defined as a disease that requires regular monitoring or therapy

^1^Cantons of Switzerland: AG Aargau, BL Basel-Land, BS Basel-Stadt, JU Jura, SO Solothurn

### Sensitivity and specificity of the ELISA test

To be able to assess sensitivity and specificity of the ELISA test we tested one matched negative specimen for each positive or borderline specimen with line blot. Of 138 specimens positive by line blot, 86 samples were positive by ELISA, resulting in a sensitivity of 62.3%. Of 613 samples negative by line blot, 584 were negative in the ELISA screening, resulting in a specificity of 95.2%.

Considering that 6.7% of the randomly selected ELISA negative tests were tested positive in the line blot analysis, we extrapolated this proportion of ELISA false-negatives to the total ELISA negatives (*n* = 780) which increased the positive samples according to scenario 1 from 128 to 180 and resulted in a seroprevalence of 18.7% (95% CI: 16.2–21.3). According to scenario 2, the corrected number of positive samples is 160 resulting in a seroprevalence of 16.6% (95% CI: 14.4–19.1).

### Cross-reaction with syphilis

Samples of 5 children were positive for *B. burgdorferi* antigens and also positive for the TpN17 band in the line blot. Syphilis serology was then performed and negative in all of them.

## Discussion

This is the first study in Europe that uses left over samples to determine current seroprevalence of *B. burgdorferi* IgG in children and adolescents in an area endemic for Lyme borreliosis. We found a seroprevalence proportion higher than 10% which is markedly higher than in other previous European studies in the same age groups where it ranged from 3.2 to 4.1% [8–10]. Even in the more conservative approach of scenario 2, with a seroprevalence of 11.2%, this proportion is still higher than in other studies in children and more similar to the range of the seroprevalence described for adults [7, 19, 20]. The seroprevalence of *B. burgdorferi* IgG with scenario 1 is comparable to the overall seroprevalence proportion of 14.5% (95% CI: 12.8–16.3) in Central Europe, as recently shown in a systematic review [4].

One explanation of the higher seroprevalence in this study is that the previous evidence was generated more than 10 years ago when the climatic conditions were different in several aspects [21]. In recent years, higher average temperatures and longer summer and autumn periods have been recorded in Europe and elsewhere. This leads to a changed behavior with children and adolescents spending more time outdoors today and therefore an increased chance to acquire tick bites. In addition, it has been documented that Swiss children spend more time in nature than their counterparts in four other European countries [22], which could also have influenced the higher seroprevalence. Since only few specimens derived from patients residing in nearby areas of France and Germany, no meaningful comparisons with those from Switzerland were possible.

Surprisingly we were not able to demonstrate an age-dependent seropositivity. Contrary to what was observed in previous studies, the seroprevalence in our study remained similar with increasing age. Considering that in Switzerland young children probably spend more time outdoors than school-age children and adolescents, a relatively high seroprevalence in the young age groups can be explained. Furthermore, Böhm et al. in a study in Germany found that reversion of seropositivity occurred in 43% of children over a period of 11 years and in particular young children were more likely to revert to seronegative over time [10]. These two factors might have contributed to a relatively stable seroprevalence throughout childhood in our study.

There was a trend towards a higher proportion of seropositive samples over the course of the year, peaking in the months of November and December. An IgG response to *B. burgdorferi* can usually be measured about six to eight weeks after infection by a tick-bite [23]. This suggests that the higher seropositivity of samples measured in November and December can be explained by accumulation of infections during the warm summer season.

In this study, residency had no effect on seroprevalence and specifically no association of urban versus rural residency was noted. This can be explained by the fact that ticks are abundant in Switzerland also in gardens and parks of urban areas and not only in the countryside. Furthermore, the regions in our study are very close together.

According to the manufacturer, the sensitivity and specificity of the ELISA compared to an immunoblot are 94.1% and 98.3%, while in our study they were 62.3% and 95.2%, respectively. Since we analyzed more than twice as many samples as the manufacturer to calculate the sensitivity and specificity, we consider our values to be accurate. Thus, a previous *B. burgdorferi* infection cannot be completely ruled out with a negative ELISA result. It is important to consider this in a clinical setting when *B. burgdorferi* infection is suspected in a child.

## Strengths and limitations

A strength of this study is that we included pediatric in- and outpatients from all clinical departments/divisions of a University Children`s Hospital with signed general consent (i.e., avoiding a selection bias) and that we were able to collect a large sample size in a short period of time. Furthermore, the age of patients was evenly distributed throughout childhood and adolescence and also sex of the specimen donors was well balanced. As a limitation, no specific questionnaires were used to assess leisure activities, time spent outdoors, and previous history of tick bites from clinical routine data was mostly not available. In addition, patients included in our study may be sicker on average than the general population, which could negatively affect the time spent outdoors. However, because no difference in seroprevalence was found between patients with or without a chronic disease, we do not suspect this to be a relevant bias. Therefore, we assume that the results of our study can be generalized to the paediatric population in our region.

## Conclusion

This study provides an important update of the estimates of IgG antibody seropositivity for *B. burgdorferi s.l.* in children and adolescents residing in North-Western Switzerland. Compared to previous studies in children, the seroprevalence of at least 11.2% in our study is markedly higher. These results confirm that *B. burgdorferi* infection is common in children and adolescents and may point towards an increased risk of infection in recent years.

## Data Availability

Data availability: Upon request from the corresponding author.
